# Functional and Molecular Characterization of *Ex Vivo* Cultured Epiretinal Membrane Cells from Human Proliferative Diabetic Retinopathy

**DOI:** 10.1155/2013/492376

**Published:** 2013-10-01

**Authors:** Zoltán Veréb, Xhevat Lumi, Sofija Andjelic, Mojca Globocnik-Petrovic, Mojca Urbancic, Marko Hawlina, Andrea Facskó, Goran Petrovski

**Affiliations:** ^1^Stem Cells and Eye Research Laboratory, Department of Biochemistry and Molecular Biology, Medical and Health Science Center, Faculty of Medicine, University of Debrecen, Debrecen H-4010, Hungary; ^2^Eye Hospital, University Medical Centre Ljubljana, SI-1000 Ljubljana, Slovenia; ^3^Department of Ophthalmology, University of Szeged, H-6720, Hungary

## Abstract

Characterization of the cell surface marker phenotype of *ex vivo* cultured cells growing out of human fibrovascular epiretinal membranes (fvERMs) from proliferative diabetic retinopathy (PDR) can give insight into their function in immunity, angiogenesis, and retinal detachment. FvERMs from uneventful vitrectomies due to PDR were cultured adherently *ex vivo*. Surface marker analysis, release of immunity- and angiogenesis-pathway-related factors upon TNF**α** activation and measurement of the intracellular calcium dynamics upon mechano-stimulation using fluorescent dye Fura-2 were all performed. FvERMs formed proliferating cell monolayers when cultured *ex vivo*, which were negative for endothelial cell markers (CD31, VEGFR2), partially positive for hematopoietic- (CD34, CD47) and mesenchymal stem cell markers (CD73, CD90/Thy-1, and PDGFR**β**), and negative for CD105. CD146/MCAM and CD166/ALCAM, previously unreported in cells from fvERMs, were also expressed. Secretion of 11 angiogenesis-related factors (DPPIV/CD26, EG-VEGF/PK1, ET-1, IGFBP-2 and 3, IL-8/CXCL8, MCP-1/CCL2, MMP-9, PTX3/TSG-14, Serpin E1/PAI-1, Serpin F1/PEDF, TIMP-1, and TSP-1) were detected upon TNF**α** activation of fvERM cells. Mechano-stimulation of these cells induced intracellular calcium propagation representing functional viability and role of these cells in tractional retinal detachment, thus serving as a model for studying tractional forces present in fvERMs in PDR *ex vivo*.

## 1. Introduction

Diabetes mellitus is a micro- and macrovascular disease which can cause a sight threatening diabetic retinopathy in up to 80% of patients having the disease for 10 or more years [1]. Based upon the Wisconsin Epidemiologic Study of Diabetic Retinopathy (WESDR), the 10-year incidence of new retinopathy was 89% in the group diagnosed before age 30 years, 79% in the insulin-taking group of 30 years or older and 67% in the noninsulin-taking group [2]. The disease itself affects those individuals who are in their most productive years; therefore, it poses great socioeconomic burden on the society [3]. Proliferative diabetic retinopathy (PDR) is an advanced stage of the ocular manifestations, hallmarked by neovascularizations and late stage fibrovascular proliferations on the surface of the retina, which can eventually lead to tractional retinal detachment causing blindness [4]. Symptomatically, PDR is accompanied by visual field defects and blurred vision, while funduscopy can reveal fibrovascular membranes and neovascularizations with cotton wool spots and flame- and dot-blot hemorrhages as well as hard exudates. 

Although the exact pathophysiological mechanism leading to PDR remains still unclear, theories about growth hormone involvement, sluggish platelet and erythrocyte circulation with consequent focal capillary occlusions and retinal ischemia, activation of aldose reductase pathway, and consequent damage of intramural pericytes that altogether cause saccular outpunching of capillaries, ruptured microaneurysms, intra- and epiretinal hemorrhages, and exudation have all been described as causes of diabetic retinopathy [5, 6]. The metabolic pathways that have been associated with this disease include activation of the polyol pathway, nonenzymatic glycosylation, and activation of the *β* isoform of protein kinase C (PKC-s) [7]. Retinal ischemia has been considered to be the trigger for production of vasoproliferative factors, which can then stimulate new vessels formation and penetration through the internal limiting membrane to form fibrovascular epiretinal membranes (fvERMs) between the retina and the posterior hyaloid face. Besides these mechanisms, high levels of proinflammatory cytokines such as interleukin 6 (IL-6), IL-8, and tumor necrosis factor alpha (TNF*α*) have been measured in samples from the vitreous body of patients with PDR [8, 9].

So far, the origin of the cells found in the fvERMs has not been well understood. Although attempts to assess the presence of CD34^+^ and CD31^+^ vascular endothelial cells have been made using histological means in postvitrectomy membranes [10–12], no such assessment has been made when the cells are cultivated *ex vivo* under adherent conditions. 

In the present study, we adherently cultivate the cells growing out of the fvERMs and perform surface profiling using markers for hematological, endothelial, and mesenchymal stem cells (MSCs) and cell adhesion molecules (CAMs) to determine the possible origin of these cells. Furthermore, the angiogenic potential of the fvERM outgrowing cells under presence or absence of proinflammatory factor TNF*α* is also determined using high-throughput screening by angiogenic protein array, while measurement of the intracellular calcium dynamics is performed in response to mechanostimulation to prove the viability and functionality of these cells and to mimic the tractional forces appearing due to presence of fvERMs in PDR. 

## 2. Materials and Methods

### 2.1. Tissue Collection and Cultivation of Cells

All tissue collection complied with the Guidelines of the Helsinki Declaration (1964) and was approved by the National Medical Ethics Committee of the Republic of Slovenia. FvERMs were obtained from patients (mean age: 62.7 ± 9.0 years) undergoing vitrectomy due to intravitreal hemorrhage in PDR (Table 1 shows the data for each patient). Transport and *ex vivo* cultivation under adherent conditions were performed immediately after isolation in DMEM:F12 (Sigma-Aldrich, Ljubljana, Slovenia) supplemented with 10% fetal calf serum (FCS) (PAA Laboratories GmbH, Pasching, Austria) and kept until reaching confluence. Primary human retinal pigment epithelial (hRPE) cells were isolated from cadavers and cultivated *ex vivo* (protocol modified from Thumann et al. [13]) upon approval by the State Ethical Committee in Hungary (14415/2013/EKU-183/2013 and DEOEC RKEB/IKEB 3094/2010), for comparison to the fvERM outgrowing cells.

### 2.2. Surface Marker Analysis of the fvERM Outgrowing Cells

The phenotype of the fvERM outgrowing cells was determined by flow cytometry using the following fluorochrome-conjugated monoclonal antibodies: CD11a/lymphocyte function-associated antigen 1 (LFA-1), CD14, CD18/integrin *β*2, CD29/integrin *β*1, CD34, CD44/Homing Cell Adhesion Molecule (HCAM), CD47, CD49a, CD49d, CD51/integrin *α*V, CD54, CD73, CD90/Thy-1, CD338/ATP-binding cassette subfamily G member 2 (ABCG2), CD106/vascular cell adhesion protein 1 (VCAM-1), CD166/activated leukocyte cell adhesion molecule (ALCAM), platelet-derived growth factor receptor beta (PDGFR*β*) (all obtained from Biolegend, San Diego, CA, USA), human leukocyte antigen G (HLA-G), CXCR4, CD146/melanoma cell adhesion molecule (MCAM), HLA-DR, vascular endothelial growth factor receptor 2 (VEGFR2), CD45, CD146, CD117, CD31 (all obtained from R&D Systems, Minneapolis, MN, USA), and CD105/endoglin from BD Biosciences, San Jose, CA, USA. After harvesting the cells with 0.025% trypsin-EDTA, they were washed with normal medium and twice with Fluorescence-Activated Cell Sorting (FACS) buffer. The fvERM cells were incubated with antibodies according to the manufacturers' protocol on ice for 30 min then washed again with FACS buffer and fixed in 1% paraformaldehyde (PFA)/phosphate buffered saline (PBS) and analyzed within 1 day. The samples were measured by FACSCalibur flow cytometer (BD Biosciences, Franklin Lakes, NJ) and the data analyzed using FlowJo (TreeStar, Ashland, OR) software. The results were expressed as means of positive cells (%) ±SEM. Hierarchical clustering was performed by R software [14]. 

### 2.3. Secretion of Angiogenic Factors by fvERM Outgrowing Cells

The expanded fvERM cells were plated onto 6-well plates at a density of 2 × 10^5^ cells per well in triplicates. After 24 hrs, the medium was changed, and the cells were treated with 100 ng/mL recombinant human TNF*α* (Preprotech, Rocky Hill, NJ, USA) for additional 24 hours. The cells were then collected for analysis of the expression of cell surface markers and their supernatants collected and pooled into one stock pretreated by 0.025 N hydrochloric acid for 15 mins at room temperature. The secreted factors were analyzed by Human Angiogenesis Array (Proteome Profiler, R&D Systems, Minneapolis, MN, USA) according to the manufacturers' protocol, and the pixel density in each spot of the array was determined by ImageJ software.

### 2.4. Calcium Dynamics in the fvERM Outgrowing Cells

The cultured fvERM outgrowing cells were loaded with acetoxymethyl (AM) ester of Fura-2 (Fura-2 AM; Invitrogen-Molecular Probes, Carlsbad, CA, USA), a free cytosolic calcium (Ca^2+^) sensitive dye, which was dissolved in DMSO and suspended in 1.5 mL of culture medium (final working concentration: 8 *μ*M). The Fura-2 AM loading was carried out at 37°C, 5% CO_2_ for 40 min. After loading, the cultures were washed twice for 7 min with 3 mL of the physiological saline with (in mM) NaCl (131.8), KCl (5), MgCl_2_ (2), NaH_2_PO_4_ (0.5), NaHCO_3_ (2), CaCl_2_ (1.8), HEPES (10), and glucose (10), pH 7.24. The Petri dish was then mounted onto inverted microscope, Zeiss Axiovert S 100 (Carl Zeiss, AG, Oberkochen, Germany). To test responses to mechanical stimuli, the mechanostimulation with a tip of a glass micropipette mounted on a MP-285 micromanipulator (Sutter, Novato, CA, USA) was used. Image acquisition was done with the 12-bit cooled CCD camera SensiCam (PCO Imaging AG, Kelheim, Germany). The software used for the acquisition was WinFluor (written by J. Dempster, University of Strathclyde, Glasgow, UK). Microscope objectives used were 10x/0.30 Plan-NeoFluar and 63x/1.25 oil Plan-NeoFluar (Zeiss). The light source used was XBO-75W (Zeiss) Xe arc lamp. The excitation filters used, mounted on a Lambda LS-10 filter wheel (Sutter Instruments Co.), were 360 and 380 nm (Chroma). Excitation with the 360 nm filter (close to the Fura-2 isosbestic point) allowed observation of the cells' morphology and of the changes in the concentration of the dye, irrespective of changes in free cytosolic Ca^2+^ concentrations ([Ca^2+^]i), while the 360/380 nm ratio allowed visualization of the [Ca^2+^]i changes in the cytoplasm. Image acquisition, timing, and filterwheel operation were all controlled by WinFluor software via a PCI6229 interface card (National Instruments, Austin, TX, USA). Individual image frames were acquired every 500 ms resulting in frame cycles being 1 second long (two wavelengths). 

### 2.5. Statistical Analysis

Each experiment was performed at least three times, and each sample was tested in triplicates. Statistica 7.0 software (StatSoft Inc., USA) was used for the statistical analyses. Statistically significant difference between the two groups (fvERM cells versus primary hRPE) was determined with paired student *t*-test, and a value of *P* < 0.05 was considered significant. Data are expressed as mean ± SD or SEM.

## 3. Results

### 3.1. Immunophenotyping of the fvERM Outgrowing Cells

**Figure 1:**
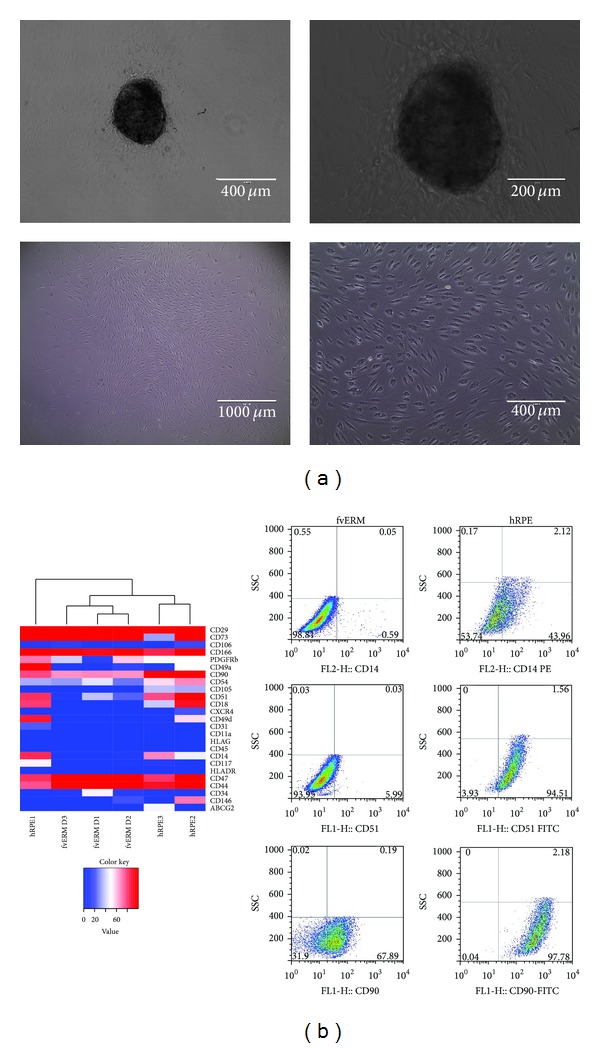
Morphology (a) and hierarchical clustering (b) based upon the expressed surface markers on fvERM and primary human retinal pigment epithelial (hRPE) cells. (fvERM D1, 2, and 3 are fvERM Donor 1, 2, and 3, resp.; data shown are representative of 3 independent experiments on 3 different donor fvERM samples).

The fvERM outgrowing cells assumed an elongated, fibroblastoid like morphology when cultivated under adherent conditions *ex vivo* (Figure 1(a)). The surface marker expression profile of the cultivated fvERM cells was compared to that of primary hRPE cells (Figure 1(b) (cluster analysis) and Table 2). The *ex vivo* cultured fvERM cells showed no purely common hematopoietic or monocytic phenotype. Similarly, these cells expressed no CD45, CD11a (LFA-1), and HLA-G, like the primary hRPE cells (an exception being the very low CD11a expression in one of the hRPE donors). A higher percentage of the primary hRPE cells were positive for CD14 (66.60 ± 11.26%) compared to the fvERM cells (1.81 ± 1.06%; *P* = 0.005), while inversely, higher CD47 expression was observed on the fvERM (97.95 ± 0.44%) compared to the primary hRPE cells (88.04 ± 5.48%)—the latter showing that the outgrowing fvERM cells were indeed viable cells. Both cell types had a low surface expression of HLA-DR (0.08 ± 0.08% in fvERM cells versus 1.00 ± 1.00% in hRPE), while the percentage of CD117/c-kit (0.94 ± 0.76% and 19.80 ± 16.53%); CXCR4 (0.41 ± 0.25% and 7.28 ± 5.22%); and CD338/ABCG2 (0.80 ± 0.08% and 17.63 ± 15.09%) cells was in general lower in the fvERM compared to the primary hRPE, respectively. Only the expression of CD34 was more abundant in the fvERM cultures (21.81 ± 15.78%) compared to the primary hRPE (2.34 ± 1.17%); however, this difference was not statistically significant. Similarly, the expression of CD73, CD105, and PDGFR*β* was not significantly different between the fvERM cells and the primary hRPEs, while a significant difference in the CD90 expression was measured between the two cell types (68.19 ± 0.46% in fvERM cells versus 91.16 ± 6.66% in primary hRPE; *P* = 0.03).

Among the cell adhesion molecules (CAMs) and integrins, all being important for maintaining the fate of the cells in their environment, significantly lower expression of CD18/integrin *β*2 (*P* = 0.01) and CD51/integrin *α*V (*P* = 0.004) was found on fvERM cells compared to primary hRPE cells. The expression of CD29/integrin *β*1, CD49a/integrin *α*1, CD49d/integrin *α*4, CD54/intercellular adhesion molecule 1 (ICAM-1), CD106/V-CAM 1, CD146/MCAM, and CD166/ALCAM on fvERM cells were similar to those detected on primary hRPEs. 

### 3.2. Detection of Angiogenic Factors Secreted by the fvERM Outgrowing Cells

**Figure 2:**
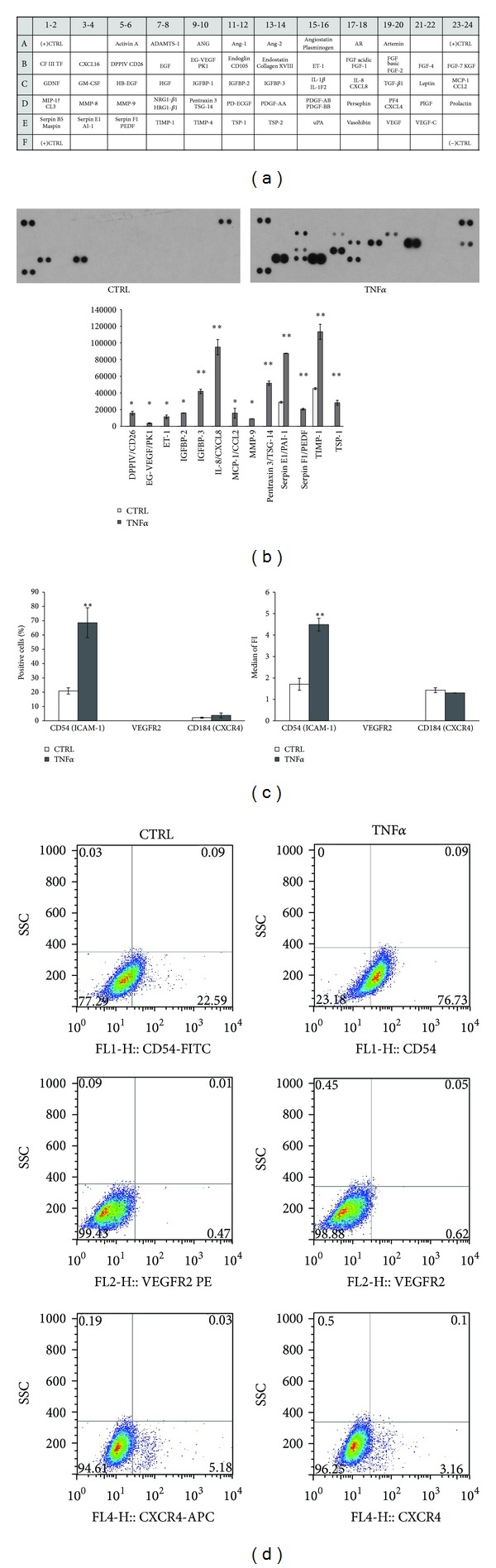
Angiogenic factors secreted by the fvERM cells. The fvERM cells were treated with 100 ng/mL TNF*α* for 24 hours. Supernatants were collected, and cytokine levels were determined by a Human Angiogenesis Array Proteome Profiler. Map of the 55 angiogenesis-related proteins detected on the membranes (a). Panel of the secreted proteins of control and TNF*α* treated cells (b). Flow cytometric surface marker analysis of the TNF*α* treated cells ((c), (d)). (Data shown are mean ± SD of 3 independent experiments on 3 different donor fvERM samples).

Altogether 55 angiogenesis-related markers were screened from the supernatants of the fvERM outgrowing cells under presence or absence of TNF*α* treatment. Unstimulated fvERM cells expressed high amount of serine protease inhibitor E1 (Serpin E1) also known as endothelial plasminogen activator inhibitor 1 (PAI-1; at pixel density 28891 ± 1096.02) and tissue inhibitor of metalloproteinase 1 (TIMP-1; at pixel density 45238.5 ± 1170.26) as shown in Figure 2. After 24 hours of proinflammatory stimulation by TNF*α*, both factors increased further in the supernatants of the treated compared to the control untreated cells (Serpine E1 with pixel density 87418.5 ± 243.24 and TIMP-1 with 113313 ± 9050.26, resp.). More importantly, 11 secreted angiogenesis-related factors could be detected in the cell culture media of fvERM cells treated by TNF*α* that were otherwise absent in the untreated controls. Besides Serpine E1, the antiangiogenic and tumorigenic pigment epithelium-derived factor (PEDF, also known as Serpin F1) was induced and secreted (at pixel density 20601.5 ± 1045.10). Endothelin 1 (ET-1; at pixel density 11427 ± 2065.46), a molecule that has been implicated in the development and progression of vascular disorders and usually secreted by endothelial cells upon stimulation by proinflammatory cytokines or hypoxia, could also be detected upon TNF*α* treatment (Figure 2). In addition, TNF*α* stimulation caused expression of 14 TNF-inducible proteins, among them being the pentraxin-related protein 3 (PTX3), which is a marker for rapid primary local activation of innate immunity and inflammation (at pixel density 51756 ± 2533.56). Monocyte chemotactic protein-1 (MCP-1 or CCL2; at pixel density 15799.5 ± 5861.92) and IL-8 (also refered as CXCL8; at pixel density 94931 ± 9130.87) were both released by the TNF*α* treated, but not the untreated, fvERM outgrowing cells; these molecules play an important role as monocyte chemoattractant proteins. High pixel density of thrombospondin 1 (TSP-1; 28239 ± 2942.27) could also be measured in the proteome profiler array of the TNF*α* stimulated fvERM cells, referring to its many angiogenic and antiangiogenic functions that depend upon its binding factor alternatives. Endocrine-gland-derived vascular endothelial growth factor (EG-VEGF)/prokineticin (PK), which is a new members of the angiogenic cytokine family, was also secreted by the TNF*α* stimulated cells, although in low amounts (at pixel density 3698 ± 627.20), and it could not be detected in the control cell culture supernatants. Dipeptidyl peptidase-4 (DPPIV also known as CD26), which plays a key role in the glucose metabolism, underwent induction upon TNF*α* treatment (at pixel density 15854.5 ± 2201.93). In addition, two members of the insulin-like growth factor-binding proteins (IGFBPs) appeared upon the TNF*α* proinflammatory stimulus IGFBP-2 (at pixel density 15965.5 ± 222.03) and IGFBP-3 (at pixel density 41872.5 ± 2607.81). Matrix metallopeptidase 9 (MMP-9), which has many biological functions, among them being facilitation of angiogenesis upon inflammatory stimulation, also increased upon TNF*α* treatment (at pixel density 8989.5 ± 134.35), while it was absent in the supernatants from the control fvERM cells. In correlation to the FACS surface immunophenotype of the fvERM cells, TNF*α* treatment increased the percentage of CD54/ICAM-1 positive cells within the cell cultures and the amount of the surface protein as well (Figures 2(c) and 2(d)) but had no influence on the VEGFR2 or CXCR4 expression, indicating that these cells do not participate directly in the angiogenesis process through endothelial differentiation.

### 3.3. Functionality and Viability of the fvERM Outgrowing Cells

**Figure 3:**
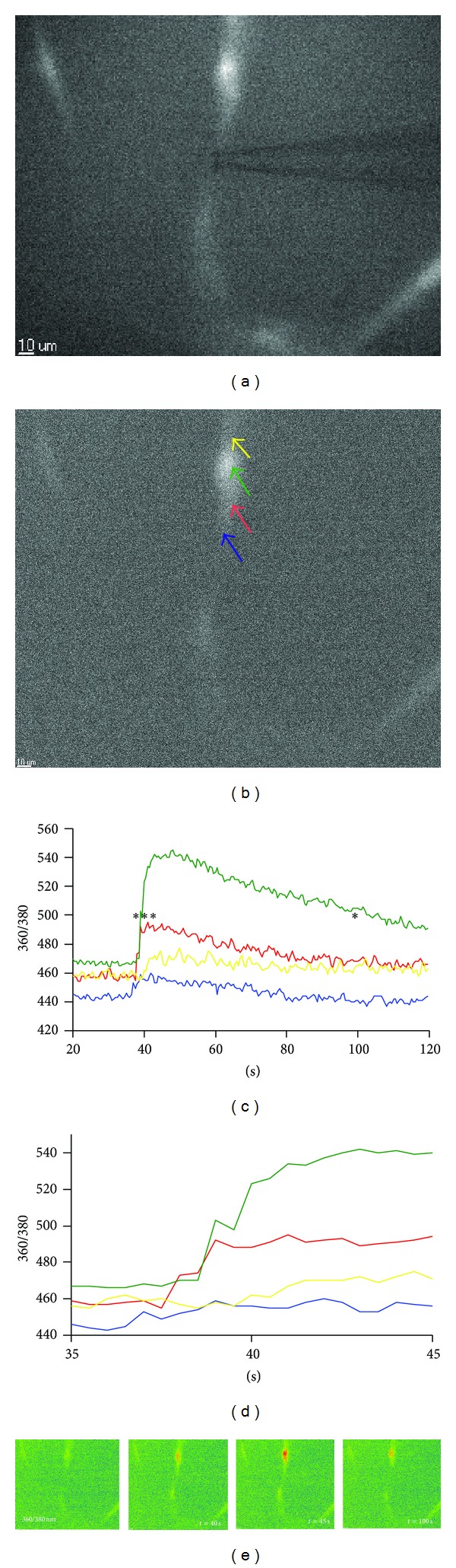
Intracellular propagation of calcium signal upon mechanical stimulation in fvERM cells from PDR. Mechanical stimulation by a glass pipette (a). Image showing the positions of the regions of interest (ROIs) presented in different colors to which correspond the traces on (b), (c), and (d). The colored traces show the intracellular calcium concentration changes in time before and after the mechanical stimulation, with different colors corresponding to the regions of the cell marked with corresponding colors (c). Enlarged region of the traces in (c) showing that calcium increase starts in the cell region marked in blue and then propagates with a time delay to the cell regions marked in red-green and then yellow (d). Color-coded images showing the intracellular calcium concentration distribution for four selected instants marked in (c) with asterisk (e).

The dynamics of [Ca^2+^]i upon mechanical stimulation reflects well upon the functionality and viability of the outgrowing fvERM cells, and such mechanical tractional forces can be common in fvERMs in late stages of PDR [15]. Mechanostimulation was induced by a glass micropipette applied to a single cell (Figure 3(a)), which caused intracellular calcium propagation that could be followed from the cell body to the periphery (Figure 3(b)). The fvERM outgrowing cells responded to mechanostimulation by increasing their [Ca^2+^]i in a monophasic manner (Figures 3(c) and 3(d)). The parts of interest are shown by colored arrows superimposed onto the morphology image. The colors correspond to the traces showing the time courses of the 360/380 ratio, proportional to [Ca^2+^]i for the selected areas. The resting levels, the increase in 360/380 ratio upon stimulation, and the amplitudes of the ratio of the responses corresponding to the resting levels and the changes in [Ca^2+^]i can all be visualized in Figure 3.

## 4. Discussion

PDR is a devastating eye disease which can lead to blindness thus needs further cellular phenotyping in order to better understand how different cells play in the formation and consequences from having fvERMs. Circulating endothelial progenitor cells (EPCs) have been shown to play a key role in the angiogenesis and neovascularization of the retina [16–18] as well as the pathology of diabetic retinopathy [19–23]. EPCs express hematopoietic stem-cell- and monocyte-surface markers such as CD31, CD34, CD45, CD14, and VEGFR2. Our fvERM and primary hRPE cells expressed CD14, CD31, and CD34 to certain levels *ex vivo*, without expressing the other known EPC or retinal endothelial cell markers [24]. The surface marker expression pattern of the primary hRPE cells appeared to be the closest to an EPC phenotype [25], although no CD45 positivity could be detected on them [26]. HLA-DR expression was very low in both fvERM and primary hRPE cells, while no HLA-G expression was detected on these cells that is in line with previous findings [27–30]. Animal studies have shown that CD117/c-kit is more likely expressed by retinal progenitor cells [31–33] and angiogenic cells [18, 34], which to a certain extent was the case with our primary hRPE, but not the fvERM cells. From the fibroblastoid or mesenchymal stem cell (MSC) markers (CD73, CD90, and CD105) [35], CD73 was present on both fvERM and primary hRPE cells. CD73 has been described as a retinal photoreceptor progenitor marker in mice [36–38] and found on human RPE cells as well [39]. Similarly, CD90 was expressed by both cell types, while CD105 did not reach a sufficient expression level to qualify the cells as MSCs. Interestingly, ABCG2 which is a well-known stem cell marker and an important player in the maintenance of stemness in retinal progenitor cells was expressed at a low level on fvERM cells (<1%), while it was much higher on the primary hRPE cells (17.63 ± 15.09%) [40].

The cell adhesion molecules (CAMs) and integrins profile are very important in cell-based tissue integrity and immune response processes. Our primary hRPE cells increased the ICAM-1 expression upon TNF*α* proinflammatory stimulus [26, 41–44], similar to the fvERM cells, meaning that these cells function as activated epithelial cells for leukocyte adhesion [45, 46]. Interestingly, no CD11a (LFA-1) positivity could be observed in both cell types under basal conditions. The cellular and soluble forms of ICAM-1 have been frequently detected in diabetic fibrovascular membranes [47], epiretinal membranes, serum or vitreous [48–51] of patients with PDR. Although CD146 has been known to be expressed on many cell types under physiological or pathological conditions, its presence and function in fvERM or primary hRPE cells has not yet been described. Our cells expressed small levels of CD146, which has generally been described as MSC marker [35], but also as novel endothelial biomarker, which plays an essential role in the angiogenesis by interacting directly with VEGFR2 found on endothelial cells [25]. In addition, CD146 has been accepted as a marker of a new EPC subset as well [26]. Although no data exists about the positivity of fvERM and primary hRPE cells for CD166, cancer-, stem-, and retinal-endothelial cells have been shown to express it [23]. The integrin pattern of our cells differed in their CD18/integrin *β*2 and CD51/integrin *α*V expression. Previously, CD29/integrin *β*1 [52], CD51/integrin *α*V, and CD44/HCAM have been described to be present on the surface of hRPE cells [45, 53, 54], but we detected more positivity for integrin *α*1 and integrin *α*2 in our primary hRPE cell cultures compared to the studies published to date [53]. Our fvERM cells expressed low levels of the *α*-subunit-containing integrins, besides a reported expression of integrin *α*4 in diabetic retinopathy [53, 55]. Interestingly, the presence of integrin *β*2 subunit has been considered important factor in the RPE-T cell interaction [56].

Protein array screening is a fast, efficient, and specific method for detecting a large number of interacting factors involved in the angiogenesis pathway. Among the factors present on our array, the PDGF receptor and its subunits have already been detected on RPE cells [57, 58] and investigated in ERMs of patients with proliferative vitreoretinopathy (PVR) [59]. The PDGFR*α* subunit has been found to be more frequent than the PDGFR*β* subunit [59, 60]. PDGF, the ligand for the PDGF receptor, is an autocrine growth factor produced by hRPE cells [61] and involved in the wound healing and migration of these cells towards wounds; the interaction of the ligand with its receptor seems to be exaggerated during wound repair and, therefore, ERM formation. Indeed, cells isolated from ERMs removed during vitrectomy for PVR show expression of PDGF receptor and are usually identified as RPE cells [24]. Others have shown that while the activation of RPE cells with IL-1*β* or TNF-*α* increases the CXCR4 mRNA expression, this increase has no effect on the percentage of cells expressing CXCR4 [62]. Our fvERM cells did not express CXCR4 upon TNF*α* stimulation, similar to how other cell types like HUVEC or Langerhans cells behave [62]. Furthermore, this may indicate that fvERM cells are not the migrating cells in diabetic retinopathy in response to increased local SDF1*α* [23], nor that cell migration occurs via the well-known SDF1*α*-CXCR4 axis. 

Presence of TNF*α* in the vitreous is an important marker for PDR [50, 63]. Cells with angiogenic potential can induce endothelial differentiation and angiogenesis. We investigated the possible expression of VEGFR2 on fvERM cells upon stimulation with TNF*α* and found no expression of this receptor under such treatment. Despite this finding, cells that are not directly involved in the angiogenesis can secrete pro- or antiangiogenic factors during cell differentiation to either support or inhibit vessel formation in their microenvironment, respectively. Our fvERM cells secreted MCP-1/CCL-2 and IL-8/CXCL-8 upon TNF*α* treatment phenomenon which has been observed in ARPE-19 cells and primary hRPE cells. Besides TNF*α*, IL-1*β*, and coculturing with T lymphocytes have all been shown to induce IL-6, IL-8, and MCP-1 secretion in ARPE-19 cells [64–66]; in addition, TLR3 ligands have been shown to have the same effect on hRPE cells [44]. MCP-1 can exhibit chemotactic activity, attracting monocytes to the site of inflammation and causing MCP-1-activated apoptosis in hRPE cells [67]. Furthermore, MCP-1 has been detected in the aqueous humor of patients with diabetic retinopathy [68], and its level were higher in a rodent model of this disease [69]. MMPs play a key role in the first stage of cell migration, connective tissue remodeling and degradation of basal lamina and surrounding extracellular matrix (ECM) during neovascularization [70]. MMP-9 appeared, while TIMP-1 levels increased upon TNF*α* treatment of fvERM cells. Although the MMP-9 level has been previously determined in PDR [71–73], nothing is known about its level in healthy retina [72]. TIMP-1 and TIMP-2 have both been measured in the vitreous of patients with PDR [74]: increased levels of TIMP-1 have been associated with the disease [72, 74–76]. Our fvERM cells expressed and secreted a basal level of TIMP-1, probably serving as a countercoup to angiogenesis via inhibiting MMPs [77]. 

Among the diverse immunological activities of PTX3, its role in innate immunity and inhibition of the uptake of apoptotic cells by macrophages and dendritic cells (DCs) have been well described [78, 79]. The induction of PTX3 by TNF*α* in ARPE-19 cells has been previously shown [80], but no such data have been shown in primary hRPE and fvERM cells. EG-VEGF/PK-1 is a novel selective angiogenic mitogen with a special prosurvival effect in different cell types [81], including endothelial cells, DCs, monocytes, and neutrophils; no reports have been made about its presence in primary hRPE and fvERM cells. Interestingly, PK1 and PK2 have been shown to induce monocyte differentiation and activation, suggesting an immunomodulatory function at local sites of inflammation [82, 83]. 

Serpin E1/PAI-1 is a principal inhibitor of tissue plasminogen activator (tPA) and urokinase (uPA) and has been shown as secreted by hRPE under hypoxic conditions [84]. Furthermore, PAI-1 could facilitate retinal angiogenesis in a model of oxygen-induced retinopathy [85]. Serpin F1/PEDF plays a very important antiangiogenic and antioxidant role in the eye [86]. It has been found in the vitreous [87] and serum [88] of patients with PDR. 

IGFBPs have been detected in the vitreous of patients suffering from neovascular ocular diseases [89]. In particular, IGFBP-2 and IGFBP-3 appear to be potent inhibitors of IGF-I and IGF-II activity in Muller cells [90] and inhibitors of cell growth of retinal endothelial cells [91]. Overexpression of Endothelin 1 (ET-1) can lead to retinal edema, degeneration, and neuronal death in animal models [92, 93]. Dipeptidyl peptidase-4 (DPP4) is an important player in glucose metabolism, but it has also been described for its role in immunoregulation and apoptosis. Thrombospondin-1 (TSP-1) is a natural inhibitor of angiogenesis. High levels of TSP-1 have been detected in drusen found in early stage age-related macular degeneration [94]. The existence of TSP-1 in the vitreous has been controversial and sometimes reported as present [95] or absent [96] in the vitreous of PDR patients.

So far, the origin of the cells found in the fvERMs has not been well understood. There is also lack of knowledge regarding the fvERMs cells specific features, including the Ca^2+^-signaling pathways. The knowledge of Ca^2+^ dynamics is important toward understanding cell biology. Mechanical force modulates a wide array of cell physiological processes. Cells from diverse tissues detect mechanical load signals by similar mechanisms but respond differently. The diversity of responses reflects the genotype of the cell and the mechanical demands of the resident tissue. It has been shown that hyperglycemia alters the tight control of intracellular calcium dynamics in retinal cells and may lead to the development of diabetic retinopathy [97]. Calcium signaling upon mechanical stimulation has been demonstrated in human corneal endothelial [98] and retinal pigment epithelial cells [99], rat retinal astrocytes, and Müller cells as well [100]. Mechanosensory function of Müller cells in the retina upon application of tractional force to the living retina showed that Müller cells responded to retinal stretch both with fast changes, as evidenced by transient intracellular calcium increases, and with slower changes in protein expression [101]. A model for Ca^2+^ waves in networks of glial cells has been studied [102] which confirms the importance of Ca^2+^ signaling measurements. It has also been shown that calcium signaling mediates mechanically induced human mesenchymal stem cell proliferation [103]. Calcium signaling is recognized as a regulator of hematopoiesis [104]. The application of shear stress to hematopoietic progenitor cells was shown to stimulate hematopoiesis [105]. Store-operated Ca^2+^ entry has been shown to be expressed in human endothelial progenitor cells [106]. [Ca^2+^]i stores are essential for injury induced Ca^2+^ signaling and reendothelialization [107].

An unwanted consequences of the appearance of fibrovascular membranes in PDR is the tractional force these membranes can raise upon the retina that can eventually lead to retinal detachment. Mechanical stimulation of the cells in the membranes or the retina can result in [Ca^2+^]i changes that can propagate through the cells as intercellular waves. Generation of such calcium waves occurs by the release of calcium from internal stores. The waves do not evoke changes in the cell membrane potential, but may constitute a pathway for extraneuronal signaling [108]. Along that line, isolated retinal ganglion cells can respond directly to mechanical deformation with pannexin-mediated ATP release and autostimulation of the P2X_7_ receptors [109]. Furthermore, stimulation of RPE cells with mechanical stress can upregulate MMP-2 and fibronectin (FN) expression through activation of the p38 pathway [110].

In summary, we could isolate and cultivate cells from fvERMs obtained from patients with PDR and show their proliferative potential. No pure hematopoietic, mesenchymal stem cells and epithelial-like phenotype could be detected on the fvERM cells. The release of proinflammatory cytokines and angiogenic molecules upon selective inflammatory stimulation with TNF*α* could also be shown *ex vivo*. In addition, the functionality and viability of the fvERM cells could be shown through an intracellular calcium dynamics upon mechanostimulation, resembling closely the tractional stimuli which occur in fvERMs in PDR.

## Figures and Tables

**Table 1:** Data of patients with proliferative diabetic retinopathy.

	Donor 1	Donor 2	Donor 3
Age (years)	54	72	62
Gender	Male	Female	Male
Type of diabetes	1	2	2
Duration of diabetes (years)	29	33	4
Insulin therapy	yes	Yes	yes
BMI	28.1	31.6	35.1
Arterial hypertension	Yes	Yes	Yes
Hyperlipidemia	Yes	Yes	Yes

**Table 2:** Immunophenotyping of the fvERM outgrowing cells and primary hRPE cells. The expression of different groups of surface markers was compared between fvERM and primary hRPE cells. The two cell types showed differential expression of CD14, CD18/integrin *β*2, CD51/Integrin *α*V, and CD90/Thy-1. (Data shown represent percentage of positive cells within the total cell culture and are representative of 3 independent experiments on 3 different donor fvERM samples, mean ± SD; *P* < 0.05*, *P* < 0.01**).

		fvERM cells	Primary hRPE cells
Hematopoietic	CD11a (LFA-1)	0.00 ± 0.00	0.00 ± 0.00
	CD14	1.81 ± 1.06	66.60 ± 11.26**
	CD34	21.81 ± 15.78	2.34 ± 1.17
	CD45	0.00 ± 0.00	0.00 ± 0.00
	CD47	97.95 ± 0.44	88.04 ± 5.48

Monocyte markers	CD117/c-kit	0.94 ± 0.76	19.80 ± 16.53
	CXCR4	0.41 ± 0.25	7.28 ± 5.22
	HLA-DR	0.08 ± 0.08	1.00 ± 1.00
	HLA-G	0.00 ± 0.00	0.00 ± 0.00
	CD338 (ABCG2)	0.80 ± 0.08	17.63 ± 15.09

MSC Fibroblast markers	CD73	98.37 ± 0.32	76.55 ± 22.76
	CD90/Thy-1	68.19 ± 0.46	91.16 ± 6.66*
	CD105/Endoglin	0.29 ± 0.29	23.10 ± 11.60
	PDGF R*β*	36.46 ± 14.11	56.59 ± 7.66

CAMs Integrins	CD18 (Integrin *β*2)	0.25 ± 0.17	69.86 ± 16.38*
	CD29/Integrin *β*1	98.34 ± 0.48	98.38 ± 1.40
	CD31/PECAM	0.00 ± 0.00	7.60 ± 6.52
	CD44/H-CAM	96.78 ± 1.06	89.03 ± 6.71
	CD49a/Integrin *α*1	6.06 ± 3.53	50.36 ± 25.67
	CD49b/Integrin *α*2	0.11 ± 0.07	49.30 ± 25.56
	CD51 Integrin *α*V	21.07 ± 9.14	85.98 ± 5.54**
	CD54/ICAM-1	32.65 ± 5.45	52.47 ± 10.83
	CD106/V-CAM 1	4.17 ± 2.60	6.43 ± 3.85
	CD146/MCAM	5.91 ± 5.69	24.58 ± 23.52
	CD166/ALCAM	95.70 ± 1.64	95.28 ± 4.47
